# Cell Type-Specific Roles of NF-κB Linking Inflammation and Thrombosis

**DOI:** 10.3389/fimmu.2019.00085

**Published:** 2019-02-04

**Authors:** Marion Mussbacher, Manuel Salzmann, Christine Brostjan, Bastian Hoesel, Christian Schoergenhofer, Hannes Datler, Philipp Hohensinner, José Basílio, Peter Petzelbauer, Alice Assinger, Johannes A. Schmid

**Affiliations:** ^1^Institute of Vascular Biology and Thrombosis Research, Medical University of Vienna, Vienna, Austria; ^2^Department of Surgery, General Hospital, Medical University of Vienna, Vienna, Austria; ^3^Department of Clinical Pharmacology, Medical University of Vienna, Vienna, Austria; ^4^Division of Cardiology, Department of Internal Medicine II, Medical University of Vienna, Vienna, Austria; ^5^Skin and Endothelial Research Division, Department of Dermatology, Medical University of Vienna, Vienna, Austria

**Keywords:** NF-kappa B signaling, inflammation, thrombosis, vasculature, coagulation, sepsis, blood cells

## Abstract

The transcription factor NF-κB is a central mediator of inflammation with multiple links to thrombotic processes. In this review, we focus on the role of NF-κB signaling in cell types within the vasculature and the circulation that are involved in thrombo-inflammatory processes. All these cells express NF-κB, which mediates important functions in cellular interactions, cell survival and differentiation, as well as expression of cytokines, chemokines, and coagulation factors. Even platelets, as anucleated cells, contain NF-κB family members and their corresponding signaling molecules, which are involved in platelet activation, as well as secondary feedback circuits. The response of endothelial cells to inflammation and NF-κB activation is characterized by the induction of adhesion molecules promoting binding and transmigration of leukocytes, while simultaneously increasing their thrombogenic potential. Paracrine signaling from endothelial cells activates NF-κB in vascular smooth muscle cells and causes a phenotypic switch to a “synthetic” state associated with a decrease in contractile proteins. Monocytes react to inflammatory situations with enforced expression of tissue factor and after differentiation to macrophages with altered polarization. Neutrophils respond with an extension of their life span—and upon full activation they can expel their DNA thereby forming so-called neutrophil extracellular traps (NETs), which exert antibacterial functions, but also induce a strong coagulatory response. This may cause formation of microthrombi that are important for the immobilization of pathogens, a process designated as immunothrombosis. However, deregulation of the complex cellular links between inflammation and thrombosis by unrestrained NET formation or the loss of the endothelial layer due to mechanical rupture or erosion can result in rapid activation and aggregation of platelets and the manifestation of thrombo-inflammatory diseases. Sepsis is an important example of such a disorder caused by a dysregulated host response to infection finally leading to severe coagulopathies. NF-κB is critically involved in these pathophysiological processes as it induces both inflammatory and thrombotic responses.

## General Links Between Inflammation and Thrombosis

The close association of inflammatory conditions and coagulatory processes has an evolutionary origin, as injuries require both an efficient blood clotting and an inflammatory immune response against invading pathogens. In this review we focus on the cellular interactions that link inflammation with thrombotic processes, while the plasmatic coagulation cascade is described elsewhere (1, 2). Platelets are the first functional elements that seal damaged blood vessels upon injury by forming aggregates and a subsequent thrombus. They are also the first immunomodulatory cells at the side of injury and inflammation, providing a functional link between host response and coagulation (3). Endothelial cells in an inactivated, quiescent state express potent inhibitors of coagulation and platelet aggregation. However, upon inflammatory stimuli they change their cellular program by expressing leukocytes adhesion molecules to facilitate their entry to sites of inflammation. In addition, they undergo a transition toward a more pro-coagulatory phenotype (4). Furthermore, chronic inflammation causes a phenotypic switch of vascular smooth muscle cells from a contractile to a synthetic phenotype, which is associated with secretion of pro-inflammatory mediators and which can finally result in a macrophage-like state (5). Other cells of the circulation and vasculature are altered by inflammatory conditions toward a pro-thrombotic state, as well. Monocytes and neutrophils contribute to coagulation by expression of tissue factor (6, 7), which is upregulated upon inflammation. Moreover, in their activated state, neutrophils are capable of expelling their DNA in conjunction with histones and other associated proteins thereby forming extracellular DNA designated as neutrophil extracellular traps (NETs), which exert antibacterial functions, but also induce a strong coagulatory response (8). Recent findings indicate that these processes are also a physiological part of an intravascular immunity especially in capillaries causing clinically unnoticed forms of micro-thrombosis that are termed immuno-thrombosis and which have the purpose of immobilizing invaded pathogens (9).

While both physiological hemostasis and immuno-thrombosis represent a normal response to traumas or invading microorganisms, any deregulation of these processes can lead to aberrant intravascular coagulation and a pathological obstruction of the blood flow, which is generally defined as thrombosis. This is often seen in acute inflammatory states, with sepsis representing a clinically weighty example, where patients suffer from anomalous systemic inflammation that is associated with alterations in blood coagulation and microvessel thrombosis in diverse organs (10). Furthermore, the interplay between endothelial cells, smooth muscle cells, platelets, and leukocytes becomes critical under chronic inflammatory conditions, which are a central cause in the pathogenesis of atherosclerosis driving vascular remodeling and plaque formation. Rupture or erosion of the plaques can then cause rapid thrombosis and occlusion of blood vessels that finally leads to myocardial infarction or stroke, the two major reasons of mortality worldwide. Therefore, understanding of the complex interaction between the distinct cell types in inflammation and thrombosis is necessary for prevention or treatment of cardiovascular diseases.

## The Transcription Factor NF-κB and its Inhibitors

**Figure 1:**
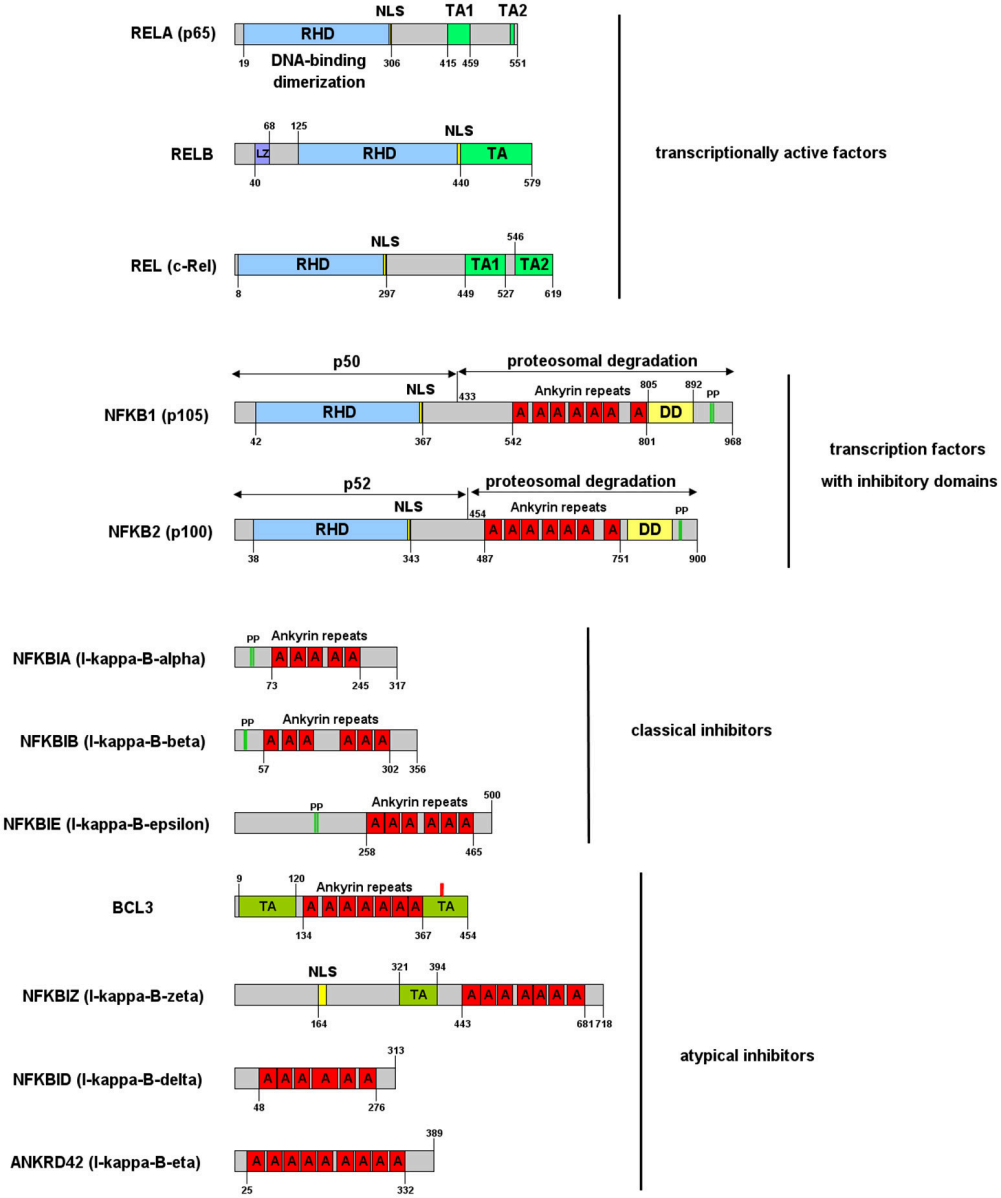
NF-κB and IκB family of proteins and their functional domains. The proteins are designated by their gene symbols with frequently used aliases in brackets. RHD, Rel-homology domain, responsible for DNA binding and dimerization; TA, transactivation domain, responsible for binding of the transcriptional machinery and RNA-polymerase; LZ, leucine zipper; NLS, nuclear localization domain; A, ankyrin repeat; DD, death domain; PP, double-phosphorylation by IκB kinases triggering ubiquitination and proteasomal degradation or processing (in case of NFKB1 and NFKB2). The numbers specify the amino acid borders of domains for human isoforms. Atypical inhibitors are described in more detail in Pettersen et al. (13).

**Figure 2:**
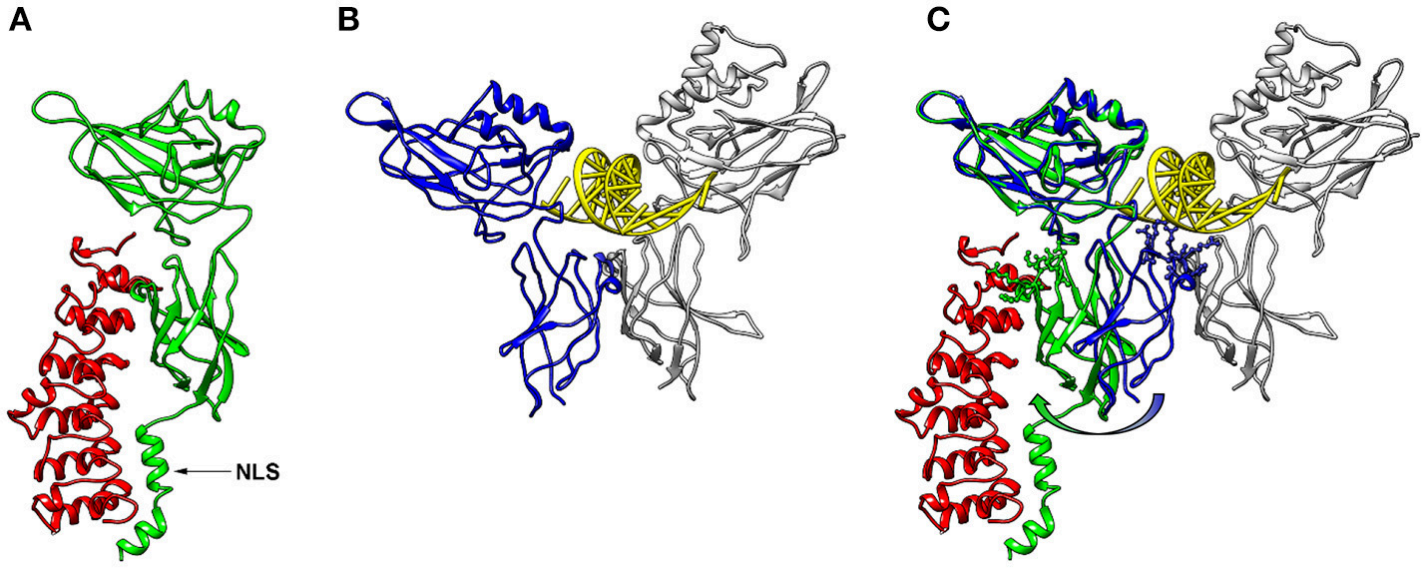
3D-structures of NF-κB/IκBα and NF-κB/DNA. **(A)** 3D-Model of a p65-NF-κB fragment (green; amino acids 20–320) bound to IκB (red, amino acids 70–282) generated with Chimera software (20) using the protein database file 1NFI. The position of the nuclear localization sequence (NLS) of p65 is indicated with an arrow. **(B)** Conformation of a p65 fragment (blue, amino acids 20–291) bound to DNA (yellow) and p50 (gray; amino acids 39–350) forming a characteristic butterfly-like structure (protein database file 1VKX). The p65-fragment, which was crystalized for this structure, lacks the last 29 amino acids of the corresponding structure of **(A)**, but is shown from the same perspective. **(C)** Superimposed structures of **(A, B)**, illustrating the conformational switch of p65 between the IκB- and the DNA-bound form (green and blue, respectively). The amino acid side chains of the lower p65 wing, which come closer than 0.5 nm to the DNA in the DNA-bound form, are shown in ball-and-stick manner. These side chains are turned away in the IκB-bound form as depicted with an arrow.

NF-κB is a central mediator of inflammation and thus fundamentally involved in the molecular links between inflammatory and thrombotic processes. It was first described in 1986 as transcription factor driving the expression of the κ-chain of immunoglobulins in B-cells (11). Thus, the commonly used abbreviation NF-κB stands for: Nuclear Factor of the κ-chain in B-cells. While the name insinuates that this protein is specific for B cells, with the κ-IgG chain being the most important target gene, it is now clear that it is expressed in nearly all cells of the human body and that it regulates the expression of hundreds or thousands of genes (12) involved in a great variety of biological processes. Not even the designation “nuclear” is correct, as this transcription factor is mostly located in the cytosol, as long as it is bound to one of its inhibitors in non-activated cells. Furthermore, NF-κB is not a single factor as implied by the name, but actually a protein family consisting of five members, building homo- or heterodimers via their Rel-homology domain, which is also responsible for DNA binding (Figure 1). Two of the family members (p100 and p105) contain inhibitory domains consisting of ankyrin repeats, which block binding to DNA and constrain nuclear localization. These have to be proteolytically processed by proteasomes for activation of NF-κB and binding to enhancer elements in the promoter regions of target genes (14–16). The processed forms of p100 and p105 (p52 and p50, respectively), do not contain a transactivation domain and need to dimerize with one of the other three family members, RelA (p65), RelB, or c-Rel to function as transcription factors. Dimers of p50 and p52 operate as transcriptional repressors, as they can bind to promoter elements without activation of the transcriptional machinery (17). The other three NF-κB proteins: p65 (RelA), RelB, and c-Rel do not contain these inhibitory domains. However, they bind to inhibitory molecules of the IκB family, which contain ankyrin repeats similar to the inhibitory domains of p100 and p105 and which have to be degraded for release and activation of the transcription factor (17). One of the most common NF-κB forms is a dimer of p65 bound to p50—the processed form of p105, with the dimer again being inactivated by association with a member of the IκB family. Binding of IκB alters the conformation of NF-κB dimers and prevents their association with DNA (18, 19) (Figure 2). Furthermore, it shifts the preferential localization from the nucleus to the cytosol. However, in contrast to the picture that is drawn in most textbooks, IκB molecules do not completely prevent translocation of NF-κB into the nucleus, as a vivid shuttling of NF-κB between cytosol and nucleus can be observed even in the presence of normal levels of IκB—with a halftime of about 7–14 min (21–23). Studies with fluorescently tagged p65 and IκB molecules in non-activated cells revealed that the concentration of nuclear p65 is about 5% of the cytosolic one (21). The basis for this phenomenon seems to be the fact that NF-κB/IκB complexes like most macromolecular complexes are subject to dissociation and re-association, with a certain number of unbound molecules under steady state conditions, which can then be recognized by the nuclear import machinery and translocated to the nucleus. As a consequence of this nucleocytoplasmic shuttling and the dynamics of binding, a low level of NF-κB activity is predicted even in non-activated cells (24). Thus, elevated levels of NF-κB molecules as observed in chronic inflammatory states can contribute to an increased risk of thrombosis even if inhibitory molecules are present.

## NF-κB Signaling Pathways

**Figure 3:**
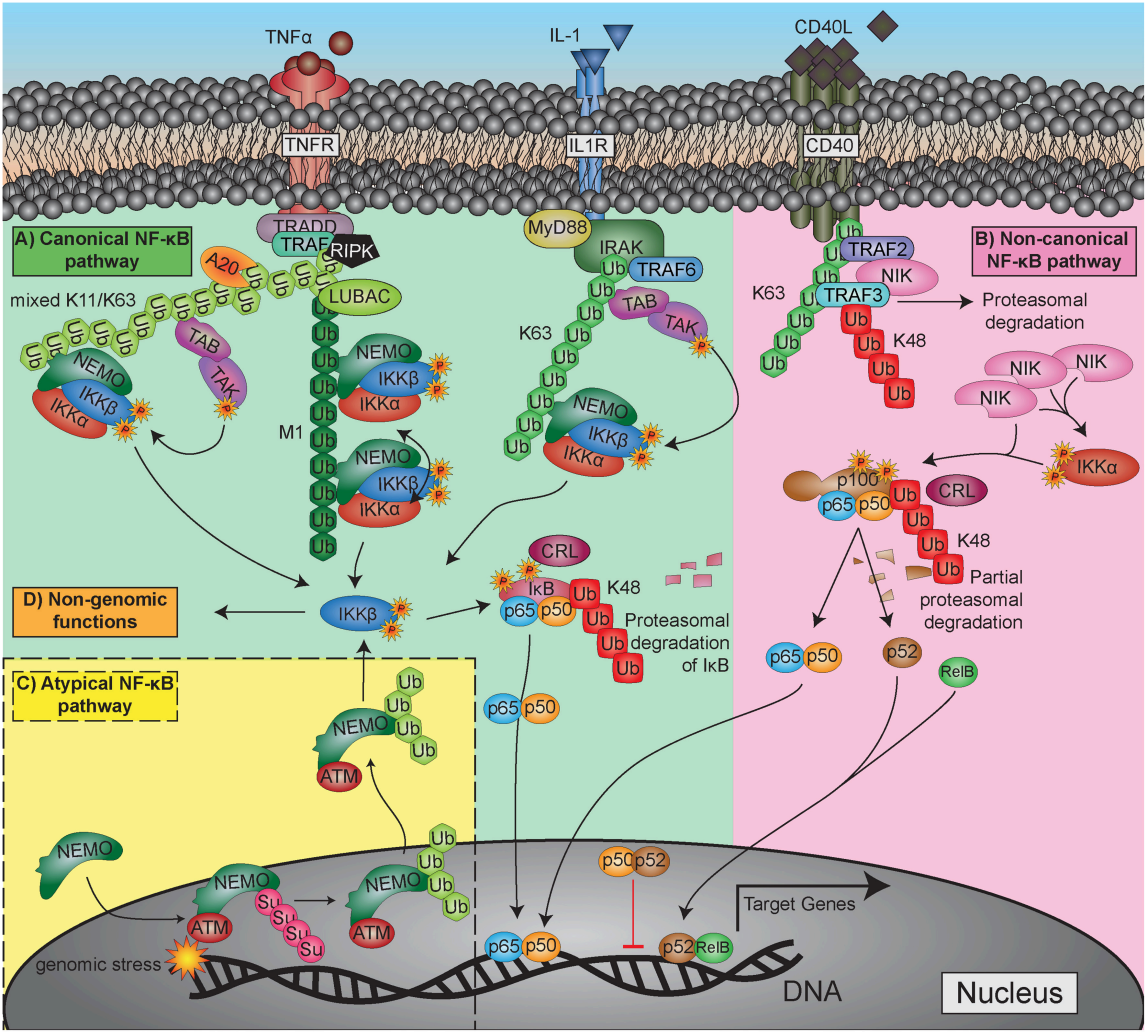
Major NF-κB activation pathways. **(A)** Canonical (classical) pathway, upper left side: exemplified by TNFα and IL-1 triggered reactions. **(B)** Non-canonical (alternative, non-classical) pathway, upper-right side: represented with the CD40L-activated pathway. **(C)** Atypical NF-κB activation pathway triggered by genotoxic stress: lower left side. For more detailed explanation see the text.

After the discovery of NF-κB as a crucial transcription factor in inflammation and immunity, great efforts have been undertaken to elucidate the signaling pathways by which it is activated. Quite soon it became clear that NF-κB activity is not only triggered by inflammatory cytokines such as tumor nerosis factor alpha (TNFα) or interleukin 1 (IL-1), but also by bacterial cell wall components like lipopolysaccharides, by viruses and even by physical stress conditions such as gamma- or UV-irradiation (see Table 1 for a more extended list of activating stimuli). The detailed clarification of the receptors that sense the original triggers and the components that transmit and modulate these signals inside the cell took many years and involved the work of numerous research groups [for a review see: (72)]. The variety of individual activation pathways became quite confusing throughout the years, so that some structuring was proposed to group the signaling cascades in a logical way. Since then, most researchers classify the activation in (i) the classical (or canonical) pathway, which is triggered by TNFα, IL-1, or lipopolysaccharide (LPS); (ii) a non-classical (non-canonical or alternative) activation elicited by CD40 ligand (CD40L) or lymphotoxin β (LTbeta); and (iii) atypical signaling pathways such as that initiated by DNA-damage (Figure 3). Yet, it has to be stated that this classification is arbitrary and should not lead to a dogmatic view of NF-κB activation. Furthermore, there appears to be a non-genomic pathway of NF-κB signaling molecules, which will be discussed in the platelet section. Moreover, it has recently been shown that stimulation of the alternative pathway can also activate components of the classical pathway and that the transcriptional responses can be qualitatively very similar (73).

**Table 1:** Important activators of NF-κB.

**Activator class**	**Examples**
Cytokines	Il-1β, TNFα (25, 26), IL-12 (27), IL-17 (28), IL-33 (29), Lymphotoxin-β (30), GM-CSF (31)
Receptor ligands	CD40L (32), BAFF [B-cell activating factor (33)], CD4-ligand [HIV-gp120, (34)], TRAIL (35), FasL (36), BMP-2 and−4 (37), EGF (38), HGF (39), insulin (40)
Bacteria	Lipopolysaccharide [LPS (41, 42)], flagellin (43), CpG-DNA (44), enterotoxins (45, 46),
Viruses	dsRNA via PKR (47), many viral proteins [as reviewed in: (48)]
Eukaryotic parasites	Candida albicans (49), Entamoeba histolytica (50), Leishmania (51)
Cell lysis products	DAMPs [Danger associated molecular patterns, (52)], HMGB1 (53), extracellular DNA(54), extracellular RNA (55, 56)
Physiological stress	ER stress (57–59), turbulent flow (shear stress) (60–62), acidic pH (63), oxidative stress (64, 65), hyperglycemia (66)
Physical stress	Ionizing radiation (67, 68), UV-light (69, 70), cold (71)
Modified proteins	Advanced glycation end products (AGEs), oxidized LDL, amyloid protein fragments

*Viruses not only activate NF-κB—but also often make use of the NF-κB pathway to control their own replication or to prevent apoptosis of host cells; furthermore, some viral genes have NF-κB binding sites and are induced by NF-κB (48)*.

Activating ligands usually trigger a conformational change or an oligomerization of receptors, which generates a binding surface for intracellular adaptor proteins. These are then recruiting E3-type ubiquitin-ligases (TRAF and IAP-proteins), which transfer a polyubiquitin chain that has been built up by E1 (ubiquitin-activating) and E2 (ubiquitin-conjugating) enzymes to target proteins such as RIPK1 or other TRAF and IAP proteins. The polyubiquitin-chains that are formed by these enzymes, are linked via lysine-63 (K63) or lysine-11 (K11), creating a structure that serves as binding and signaling platform for downstream adaptor molecules (TAB1/2 and NEMO) and their associated kinases (TAK1 and IκB kinases, IKKα, and IKKβ). Another polyubiquitination structure that activates the NF-κB signaling pathway is formed by linking the carboxy-terminus of a ubiquitin molecule with the amino-terminus of the starting methionine (M1) on the next ubiquitin moiety. This reaction is catalyzed by an enzyme complex termed LUBAC (linear ubiquitin chain assembly complex) and acts primarily on the adapter molecule NEMO or on other polyubiquitin chains as substrates (74–78). Altogether, K11-, K63-, and M1-type polyubiquitin chains generate signaling platforms via adapter molecules and clustering processes, which allow phosphorylation of IκB kinases on their activation loops. This occurs either by upstream signaling kinases such as TAK1 or by proximity-induced auto-phosphorylation in a way, that one IKK molecule activates another one in a dimer or oligomer, a process, which is commonly termed as transphosphorylation (79). Finally, most of the NF-κB-activating signaling pathways converge at the level of IκB kinases (IKKα and IKKβ), which upon activation phosphorylate the inhibitory molecules of the IκB family or the inhibitory domains of p100 and p105 on two adjacent serine residues. This triggers another type of polyubiquitination, which is characterized by linkage of ubiquitin moieties via lysine-48 (K48) and catalyzed by E3-ligases of the CRL (Cullin-Ring ligase) type, also named SCF-type (for the key components Skp1, Cullin, and F-box protein) (80, 81). These K48-linked polyubiquitin chains are recognized by proteasome activators, leading to proteasomal degradation of the inhibitor and release of the NF-κB dimer.

Important feedback mechanisms of NF-κB regulation are found at the level of the activating K63- and M1-type polyubiquitin chains. These can be degraded by specific deubiquitinating enzymes (DUBs), primarily the molecules A20 and CYLD, which act as negative regulators of NF-κB signaling. A20 or an associated E3-ligase have furthermore the capability to catalyze K48-type polyubiquitination of RIPK1, thereby leading to proteasomal degradation of this crucial NF-κB activating effector molecule (82). For a comprehensive overview of the different ubiquitination steps see Figure 3.

The biological response following NF-κB activation is manifold and depends on the cell type, the accessibility of promoter regions, which is regulated by epigenetic mechanisms, and furthermore additional feedback pathways, which intersect with the NF-κB pathway. Basically, we have to view these processes rather as dynamic signaling networks and feedback circuits far beyond the classical cascade scheme of signaling pathways involving significant crosstalk between various upstream and downstream pathways (17), which may have additional implications on the links between inflammation and thrombosis, but which are beyond the scope of this review.

**Figure 4:**
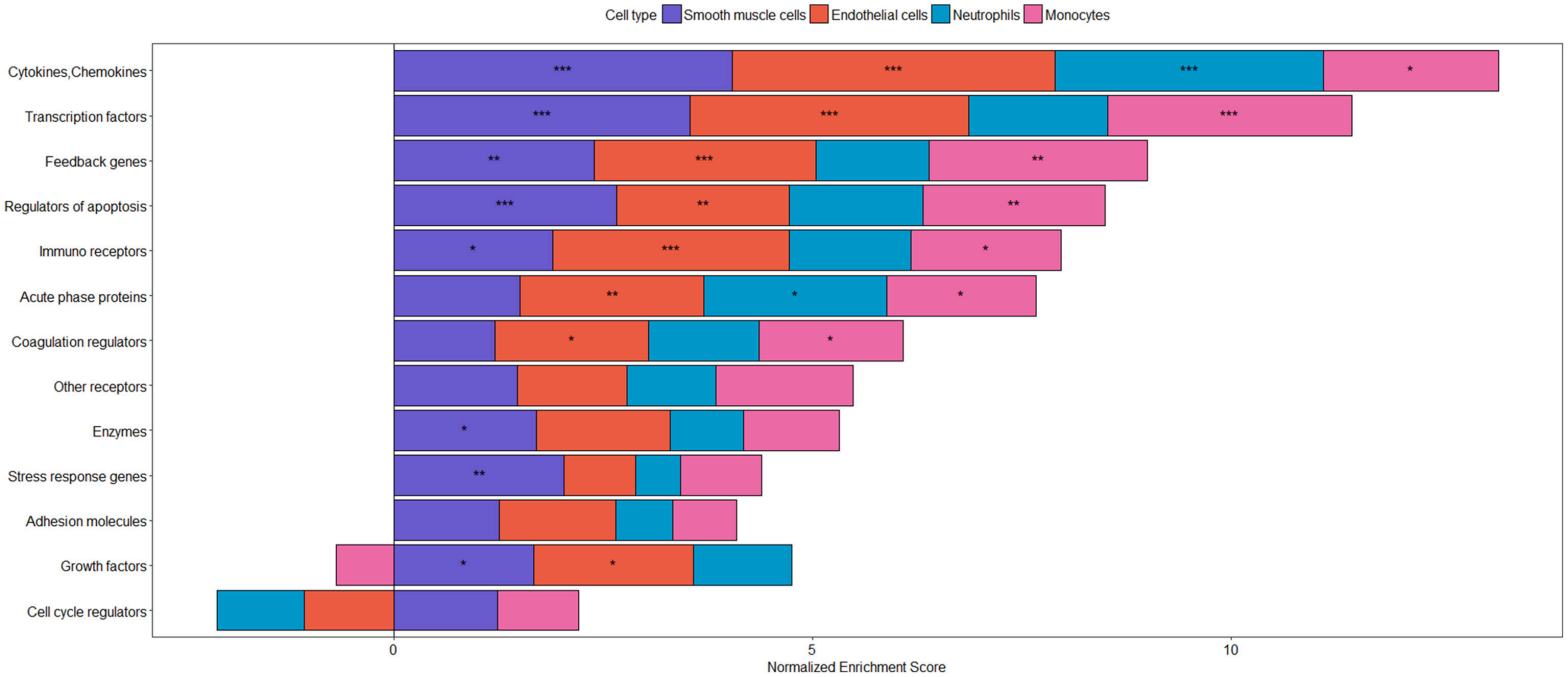
Categories of inflammatory target genes in different cell types. Transcriptional responses after stimulation with TNFα have been analyzed using gene set enrichment analysis (GSEA) with the following datasets: smooth muscle cells: GSE96962; endothelial cells: GSE96962; monocytes: GSE56681; neutrophils: GSE40548. Gene sets were derived from NF-κB target genes as described in Jia et al. (207). As *p*-value and log fold change (LogFC) are often used to evaluate significant results from differential expression analysis and the up-regulated/down-regulated genes are usually at the top and/ bottom of the ranked gene list, respectively, we used the signed z-value to rank genes, where the sign is from LogFC, as previously described (208). To assess the enrichment of the target genes of NF-kappa B gene sets in the different datasets, the GSEA Preranked tool was used (209). Gene sets showing a significant enrichment are represented by ^***^(FDR < 0.001), ^**^(FDR < 0.01), and ^*^(FDR < 0.05). The plot was produced using the R package, ggplot2 (210) visualizing the normalized enrichment scores as stacked bars showing differences in the response between different cell types of the vasculature and circulation.

The major biological function of NF-κB is to change cellular programs in all different kinds of stress situations, so that the various cell types can respond to the stress in a way that the organism can cope with the threat, activate defense mechanisms and eliminate or escape the endangering factors with the final aim to re-gain the original physiological state (83). This major biological function of NF-κB signaling explains the various types of target genes that are upregulated or induced after NF-κB activation. As listed more comprehensively in Table 2 and illustrated in Figure 4, these target genes comprise a great variety of cytokines and chemokines, the majority of which is acting in a pro-inflammatory manner, often themselves leading to NF-κB activation and thereby constituting a positive feedback circuit. This is in line with an upregulation of many different immune and chemokine receptors (211). Another set of genes that are induced by NF-κB are adhesion molecules, which are crucial for transmigration of leukocytes through the endothelium, as well as cell-cell interactions that are important for immune defense and platelet function. At the cellular level, NF-κB activation leads to upregulation of anti-apoptotic genes, which supports cell survival under stress condition. However, the same mechanism may contribute to cancer development as high levels of anti-apoptotic genes provide a survival advantage to cells with malignant mutations, which would otherwise die or become senescent (212, 213). Moreover, NF-κB induces cyclin D proteins, which are essential for cell cycle progression (214), as well as the oncogene c-Myc, which upregulates many cell cycle proteins and which is overexpressed in a majority of cancers (215). Apart from c-Myc, various other transcription factors are induced by NF-κB, such as members of the interferon-regulatory IRF family in accordance with immune defense functions, as well as HIF-1α, GATA-3 or LEF1 demonstrating that NF-κB is capable of influencing the cellular transcriptional network in a complex manner involving numerous feedback circuits (17). Additionally, NF-κB up-regulates the transcription of various members of the NF-κB gene family, thereby creating positive feedback loops. However, these are in most cases counteracted by negative feedback mechanisms, which are induced by NF-κB as well. These include the induction of the various IκB family members, which inhibit NF-κB directly, as well as proteins that are removing the activating K63- or M1-linked polyubiquitin chains from NF-κB activating proteins such as A20 or ABIN (216). Finally, the vital role of these feedback inhibitors is to shut off NF-κB activity and to revert the cell to its inactivated state. Impairment of these processes is often the basis for chronic inflammatory diseases. The complexity of all the feedback circuits is further enhanced by NF-κB-dependent upregulation of several miRNAs, which lead to the degradation or reduced translation of many different mRNAs (199).

**Table 2:** Important target genes of NF-κB.

**Target gene class**	**Examples**
Cytokines, chemokines	IL-1α and -β (84, 85), IL-2 (86), IL-6 (87), IL-8 (88), IL-12 (89), IL-17 (90), TNFα (91), IFNβ (92), IFNγ (93), CCL5 (94), Fractalkine (95), Gro (96)
Immune receptors	CCR5 (97), CCR7 (98), CD3 (99), CD23 (FcεRII) (100), CD40 (101), CD137 (102), MHC I (103), Nod2 (104), TCR (105), TLR9 (106), TNFR2 (107), TREM-1 (108)
Other receptors	A2A adenosine receptor (109), adrenoceptor α2B (110), EGFR (111), RAGE (112)
Adhesion molecules	E-selectin (113), ICAM-1 (114), fibronectin (115), P-selectin (116), VCAM-1 (117)
Acute phase proteins	CRP (118), PTX3 (119), serum amyloid A (120)
Coagulation regulators	Tissue factor [F3, (121)], F VIII (122), uPA (123), PAI-1 (124)
Anti-apoptotic genes	A20 (125), A1 (126), Bcl-2 (127), c-FLIP (128), c-IAP1 and−2 (129), BIRC3 (130), XIAP (131), TRAF1 and TRAF2 (129, 132)
Cell cycle regulators	Cyclin D1−3 (133–135), c-Myc (136)
Enzymes	COX-2 (137), lipoxygenase (138), iNOS (139, 140), nNOS (141), BACE-1 (142), cathepsin B (143), MMP-1 (144), MMP-3 (145), MMP-9 (146), GSTP-1 (147), G6PD (148), granzyme B (149), HO-1 (150), lysozyme (151), PI3K catalytic subunit (152), PKA-α (153), PKC-δ (154), PLA-2 (155), PLC- δ (156), TERT (157), transglutaminase (158)
Stress response genes	Hsp90A (159), superoxide dismutase (160, 161), Ferritin H (162)
Growth factors	FGF8 (163), G-CSF (164), M-CSF (165), GM-CSF (166), NGF (167), EPO (168), IGFBP-1 and−2 (169, 170), osteopontin (171), VEGF-C (172)
Transcription factors	AR (173), c-Myc (136), c-Rel (174), GATA-3 (175), HIF-1α (176), IRF-1 and−2 (177), IRF-4 (178), IRF-7 (179), LEF1 (180), NFKB1 (181), NFKB2 (182), Nurr1 (183), p53 (184), RelB (185), Stat5a (186)
Feedback genes	Neg. feedback: IκBα (187), IκB-ε (188), NLRP2 (189), A20 (125, 190), ABIN-1 and−3 (191),
	Pos. feedback: XIAP (131, 192), NF-κB transcription factors as mentioned above.
miRNAs	miR-9 (193), miR-21 (194), miR-143 (195), miR-125b and miR-155 (196), miR-146(197), miR-224 (198), for review see (199)
Viral genes	Adenovirus E3 (200), CMV (201), EBV (202), HBV (203), HSV-proteins (204), HPV-16 (205), SV-40 (206)

Important NF-κB target genes in the context of inflammation include various enzymes such as cyclooxygenases and lipoxygenases catalyzing the formation of prostaglandins and leukotrienes, as well as NO synthases, which are important for vasodilation and blood pressure regulation (for references see Table 2).

Furthermore, a variety of coagulation factors and regulators are induced by NF-κB, including tissue factor [F3, (121)], factor VIII (122), urokinase-type plasminogen activator (uPA) (123), and Plasminogen activator inhibitor-1 (PAI-1) (124). Thus, NF-κB contributes to coagulatory events not only via cellular activation processes, but also by transcriptional induction of proteins of the plasmatic coagulation cascade. This provides another molecular explanation for the functional links between inflammation and thrombotic processes that contributes to increased cardiovascular risk in situations of acute or chronic inflammation.

## Platelets as Mediators Between Inflammation and Thrombosis

**Figure 5:**
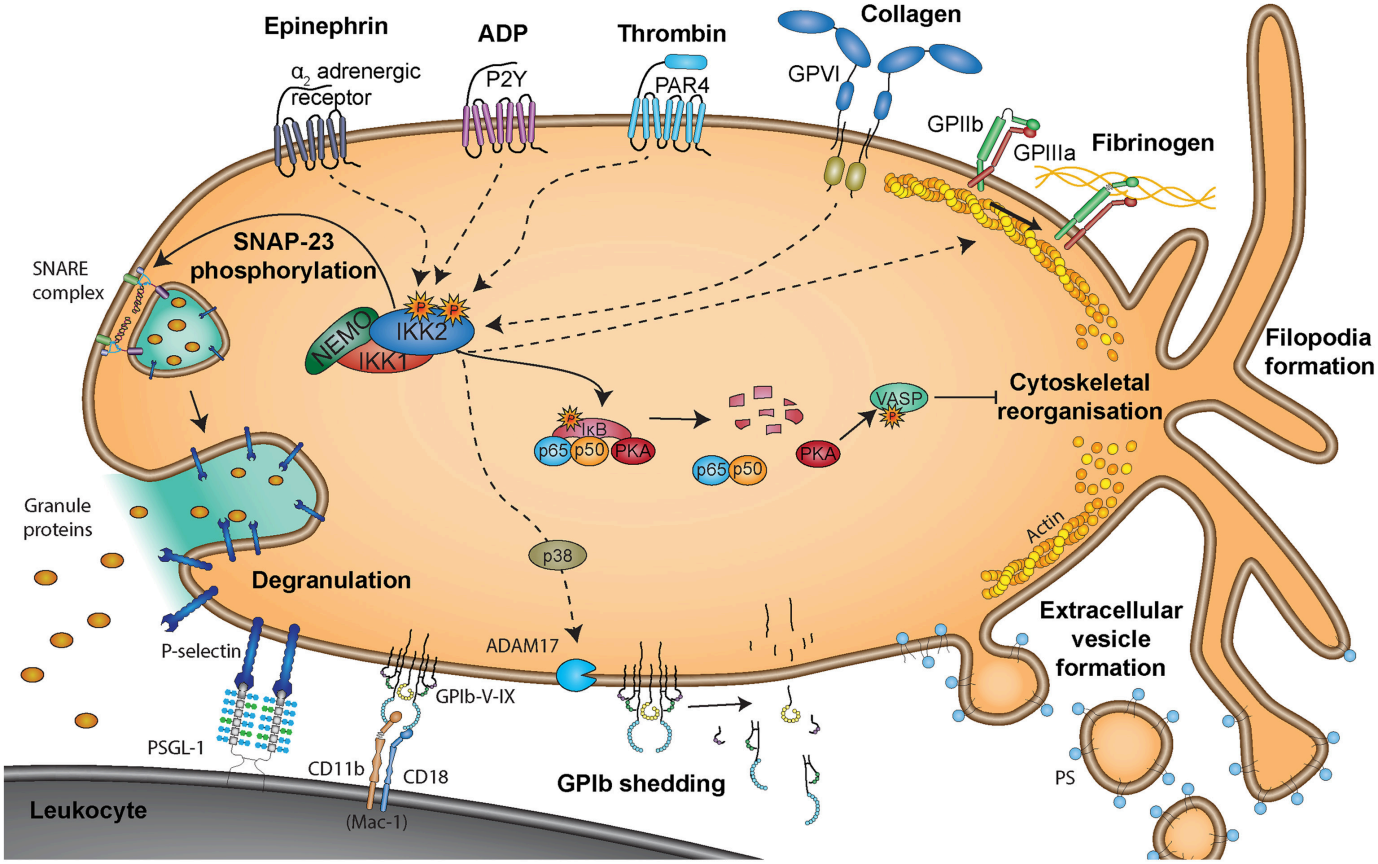
Non-genomic roles of NF-κB signaling molecules in platelets. Non-genomic effects of NF-κB signaling molecules are triggered via binding of epinephrine to α_2_ adrenergic receptors, ADP to P2Y receptors, thrombin to PAR4 receptors, collagen to glycoprotein VI (GPVI) receptors or fibrinogen to GPIIb/GPIIIa receptors. Degranulation is reported to be mediated via phosphorylation of SNAP-23 by IKK2 (251), representing a positive effect of NF-κB signaling on platelet activation. However, PKA was reported to be present in a complex with NF-κB and IκB and uncoupling of this complex upon IKK2 activation resulted in protein kinase A (PKA) activation, causing phosphorylation of vasodilator-stimulated phosphoprotein (VASP) and inhibition of platelet activity (250). Interaction of platelets with leukocytes is mediated via binding of platelet P-selectin, exposed upon degranulation, to leukocyte PSGL-1, which is supported by platelet GP-Ib-IX binding to Mac-1 on leukocytes.

Platelets, the cells that build the thrombus in primary hemostasis, are now considered crucial immune-modulatory cells providing essential functional links between inflammatory and thrombotic processes. They are small anucleate cell fragments derived from megakaryocytes with a diameter of 2–4 μm and circulate in the blood for ~7–10 days, where they patrol the endothelial wall, recognizing structures representing vessel damage. Since their discovery by Bizzozero in 1882 they are recognized for their central role in hemostasis (217), preventing blood loss upon injury by formation of platelet-platelet aggregates, which are stabilized by fibrin fibers that are formed by the plasmatic coagulation cascade (218, 219). Negative charges on the surface of activated platelets, which expose phosphatidylserine upon activation-dependent membrane lipid flip-flop, allow for calcium binding and provide the ideal surface for site-specific proteolytic activation of coagulation factors (Figure 5).

More and more evidence emerges, that activated platelets not only trigger recruitment and activation of further platelets to the site of injury but that platelets also interact with leukocytes, thereby orchestrating immune responses and mediating wound healing and repair processes via interaction with the endothelium (220–222). Activated platelets and microvesicles bind leukocytes, which leads to mutual activation and rapid, local release of platelet-derived cytokines. Platelets enhance leukocyte extravasation, differentiation and cytokine release. They propagate monocyte differentiation into macrophages and modulate oxidative burst in neutrophils [reviewed in (223)]. Toll-like receptor 4 (TLR-4)-activated platelets bind to neutrophils and initiate neutrophil extracellular trap NET formation (224). Platelets mediate NET formation either via P-selectin-PSGL1 interactions (225), neutrophils integrin αLβ2 [LFA-1 (CD11a/CD18)] (226) or platelet GPIbα (227) resulting in increased bacterial clearance. Additionally, the platelet release products thromboxane (TXA2), platelet factor 4 (CXCL4), von Willebrand factor (vWF) (228), and High mobility group box 1 (HMGB1) (229) trigger NET formation. Activated platelets and platelet microvesicle further present HMGB1 to neutrophils and commit them to autophagy and NET generation, thereby potentially causing thrombo-inflammatory lesions (229–231). Additionally, cleavage of IL-1β by NLRP3-mediated activation of caspase-1 contributes to platelet activation (232) and is associated with acute thrombotic events during hypoxic conditions (233).

Platelets can be activated by vessel injury (e.g., immobilized vWF or collagen exposure) as well as thrombin, which is generated by an activated coagulation cascade. Platelets further release substances that enhance their activation e.g., adenosine diphosphate (ADP) and TXA2. However, more and more evidence emerges that also inflammatory triggers are able to activate platelets. Platelets also express functional TLRs, including TLR2, TLR3, TLR4, TLR7, and TLR9 (234–236). Binding of LPS to platelet TLR4 induces platelet activation (237) to promote microvascular thrombosis (238) as well as platelet and neutrophil sequestration into the lung, liver and spleen (239) as well as formation of NETs.

Upon activation, platelets release their granule content, which comprises over 300 factors, involved in a plethora of processes (221). Platelet dense granules contain ADP, adenosine triphosphate (ATP), serotonin and calcium ions, which are important for activation and recruitment of further platelets. Platelet α-granules contain VWF, Factor V, and Factor VIII and fibrinogen, which can further boost activation of the coagulation cascade. Other α-granule-derived molecules like CXCL4/PF4, chemokine (C-C motif) 4 (CCL4/MIP-1), chemokine (C-C motif) 5 (CCL5/RANTES), CD40L, and P-selectin (CD62P) recruit and/or activate leukocytes, while additional factors such as vascular endothelial growth factor (VEGF), platelet-derived growth factor (PDGF) and transforming growth factor (TGF-β), act on endothelial cells and trigger angiogenesis and wound repair processes (220, 240).

Platelet granule exocytosis, which occurs via fusion of the granule membrane with the plasma membrane, involves a complex interplay of actin polymerization and proteins of the SNARE family (soluble N-ethylmaleimide-sensitive-factor attachment protein receptors), which reside on vesicles (v-SNAREs) and target membranes (t-SNAREs) (241). Synaptosomal-associated protein 23 (SNAP-23), a t-SNARE, is required for release from all three types of granules in platelets (241).

Despite the lack of a nucleus, platelets contain a variety of transcription factors as well as upstream signaling molecules and emerging evidence suggests that these factors trigger non-genomic effects in platelets rather than representing remnants of megakaryocytic packaging. Platelets are further able of shuttling transcription factors to other cells via shedding off transcription factor-laden microvesicles (242), which fulfill various effector functions (243).

Platelets contain the majority of NF-κB signaling proteins (244–249) and activation of the NF-κB/IKK/IκB pathway can be detected in response to platelet stimulation (245, 248–250) (Figure 5). While ADP, collagen, epinephrine, and thrombin all result in NF-κB pathway activation via phosphorylation of IκB and its proteasomal degradation (252), platelet activation in response to arachidonic acid does not seem to involve NF-κB (249). The precise signaling pathways contributing to NF-κB activation in platelets are currently unknown. In thrombin-activated platelets, activation of IκB kinases can be prevented by a neutral sphingomyelinase inhibitor or a p38 MAPK inhibitor downstream of the thrombin receptor protease activated receptor 4 (PAR4) but not PAR1 (253), indicating that these signal mediators are important for distinct pathways of NF-κB activation.

The effects of NF-κB, IκB and IKK on platelet activation were evaluated *in vitro* and *in vivo* using genetic ablation or inhibition of different factors of the NF-κB complex. However, these studies do not provide a conclusive picture, so far. Platelets are sensitive to NF-κB inhibitors, but the functional role of NF-κB in platelets is currently still incompletely understood. *In vivo* experiments revealed, that LDLR knockout-out mice with a platelet-specific genetic ablation of IKKβ show increased neointima formation and enhanced leukocyte adhesion at the injured area due to decreased platelet GPIbβ shedding and prolonged platelet-leukocyte interactions (254). However, another study using IKKβ-deficient platelets postulated that these platelets are unable to degranulate, leading to reduced reactivity and prolonged tail bleeding, which was postulated to be caused by defective SNAP-23 phosphorylation in absence of IKKβ (251).

*In vitro* studies using pharmacological inhibitors of IKKβ indicated that NF-κB is involved in the activation of platelet fibrinogen receptor GPIIb/IIIa (249), which is important for platelet aggregation and that the NF-κB pathway further participates in lamellipodia formation, clot retraction and stability (249). Inhibition of IKKβ and thus IκBα phosphorylation by BAY-11-7082 or RO-106-9920 suggested a positive role for IKKβ in thrombin- or collagen-induced ATP release, TXA_2_ formation, P-selectin expression and platelet aggregation (248, 249). Other studies using the NF-κB inhibitor andrographolide were in line with a positive role of NF-κB for platelet activation (255, 256) and it was also reported that platelet vitality may depend on NF-κB, as inhibition with BAY 11-7082 or MLN4924 led to depolarization of mitochondrial membranes, increased Ca^2+^ levels and ER stress induced apoptosis (257). However, in general it has to be stated that the use of pharmacological inhibitors in platelet function studies *in vitro* may suffer from artifacts of the assay system, such as inappropriate drug concentrations, which induce off-target effects, or unspecific side effects. It has been reported for instance that the commonly used IKKβ inhibitor BAY-11-7082 can induce apoptosis independent from its effect on NF-κB signaling (258) and that it is an effective and irreversible broad-spectrum inhibitor of protein tyrosine phosphatases (259).

Interestingly, NF-κB activation via IKKβ was also reported to initiate a negative feedback of platelet activation, as the catalytic subunit of PKA is associated with IκBα, from where it is released and activated when IκBα is degraded, followed by the known inhibitory actions of PKA such as VASP phosphorylation (250). This is in line with another report, where NF-κB inhibition in collagen- or thrombin-stimulated platelets led to increased VASP phosphorylation (260). With respect to the role of platelets, certainly further studies are warranted to determine, if increased levels or activity of NF-κB result in increased platelet reactivity and furthermore, how systemic chronic inflammation may affect platelet function differently than the plasmatic phase of coagulation. In general, a better understanding of NF-κB-dependent platelet responses would be crucial to fully understand the effect of NF-κB inhibitors, which are currently used as anti-inflammatory and anti-cancer agents, as they may elicit unintended effects on platelet functions.

## Megakaryocytes as Precursors of Platelets

While it is clear that platelets contain basically all upstream signaling molecules of the NF-κB pathway, as well as the transcription factors themselves, they can only respond to inflammatory triggers in a non-genomic manner. In contrast, megakaryocytes (MKs), their progenitors, can convert systemic or local inflammatory conditions to a transcriptional response, which may has consequences on the phenotype of released platelets. Megakaryocytes reside in the vascular niche of the bone marrow where they can sense inflammatory conditions via different receptors, such as TLRs and from where they release platelets into the blood circulation. Interestingly, a recent report has provided evidence that megakaryocytes are also located in the microcirculation and the extravascular space of the lung, contributing up to 50% of the total platelet production (261). At least in the bone marrow, hematopoietic stem cells undergo a unique and remarkable maturation and differentiation process to become megakaryocytes, which involves extensive endomitosis (262, 263). As a result megakaryocytes have a ploidy of up to a 128-fold chromosome-set in one single, giant, poly-lobulated nucleus (264–266), giving megakaryocytes their name. A second distinct feature of megakaryopoiesis is the generation of a complex membrane system, called demarcation membrane system (DMS) or invaginated membrane system (IMS) (264, 267–269), that serves a reservoir for later platelet production (268, 270). The final phase of megakaryocyte maturation includes the formation of proplatelets, in which long branches extend into sinusoidal capillaries allowing proplatelet release into the blood stream. The main driving force of proplatelet elongation is microtubule sliding (271). Finally, due to blood flow, platelets fission from the tips of proplatelets and are released into the blood stream (272). After transfer of the megakaryocyte's cytoplasm and DMS/IMS into platelets, the remaining denuded nucleus is removed by macrophages (273). Interestingly, it seems that apoptosis is a physiological evet for mature megakaryocytes and that peak proplatelet and platelet production is shortly followed by apoptosis (274–276).

Inflammatory cytokines and pathways are involved in various steps of megakaryopoiesis and thrombopoiesis. Megakaryocytes express toll-like receptors (TLRs) (277, 278), tumor necrosis factor receptors (TNFR1 and 2) (279), receptors for IL-1β (280, 281), and IL-6 (282, 283), all of which are important activation pathways of NF-κB. Activity of the IKK complex increases during megakaryopoiesis and decreases during thrombopoiesis, allowing controlled cell death (284). The most important signaling molecule driving differentiation and maturation of megakaryocytes is thrombopoietin (TPO), a glycoprotein primarily produced by liver and kidney. Binding of this protein to its receptor c-Mpl on bone marrow cells is the primary signaling event that promotes and regulates megakaryopoiesis (264, 285, 286). Other cytokines that synergize with TPO include IL-1α, IL-1β, IL-3, IL-6, IL-9, IL-11, and granulocyte-macrophage colony-stimulating factor (GM-CSF) (287–291). However, all of them are dependent on TPO to exert their pro-megakaryopoietic functions (291). Furthermore, immature MKs themselves express IL-1α, IL-1β, IL-3, IL-6, and GM-CSF to stimulate their ploidy via NF-κB and TPO (287–289, 292).

A further link between inflammation and megakaryopoiesis is provided by reactive oxygen species (ROS), which after being released by activated macrophages and neutrophils commit hematopoietic stem cells toward the megakaryocytic lineage (293). Interestingly, a stem cell population was identified, which is already committed to the megakaryocytic lineage and matures rapidly upon inflammatory conditions, to replenish the loss of platelets (294).

One of the most intriguing recent findings was that upon acute inflammation IL-1α leads to rapid, TPO-independent platelet production. IL-1α signaling reduces plasma membrane stability, dysregulates tubulin expression and proplatelet formation, ultimately triggering megakaryocyte rupture and release of enormous amounts of platelets within short time. In this way, platelet loss due to acute injuries, blood loss or infection can be rapidly compensated (281).

To conclude, it can be stated that inflammation in general and NF-κB signaling in particular, does not only directly affect platelets, but also indirectly via modulation of their megakaryocytic progenitors.

## Endothelial Cells

**Figure 6:**
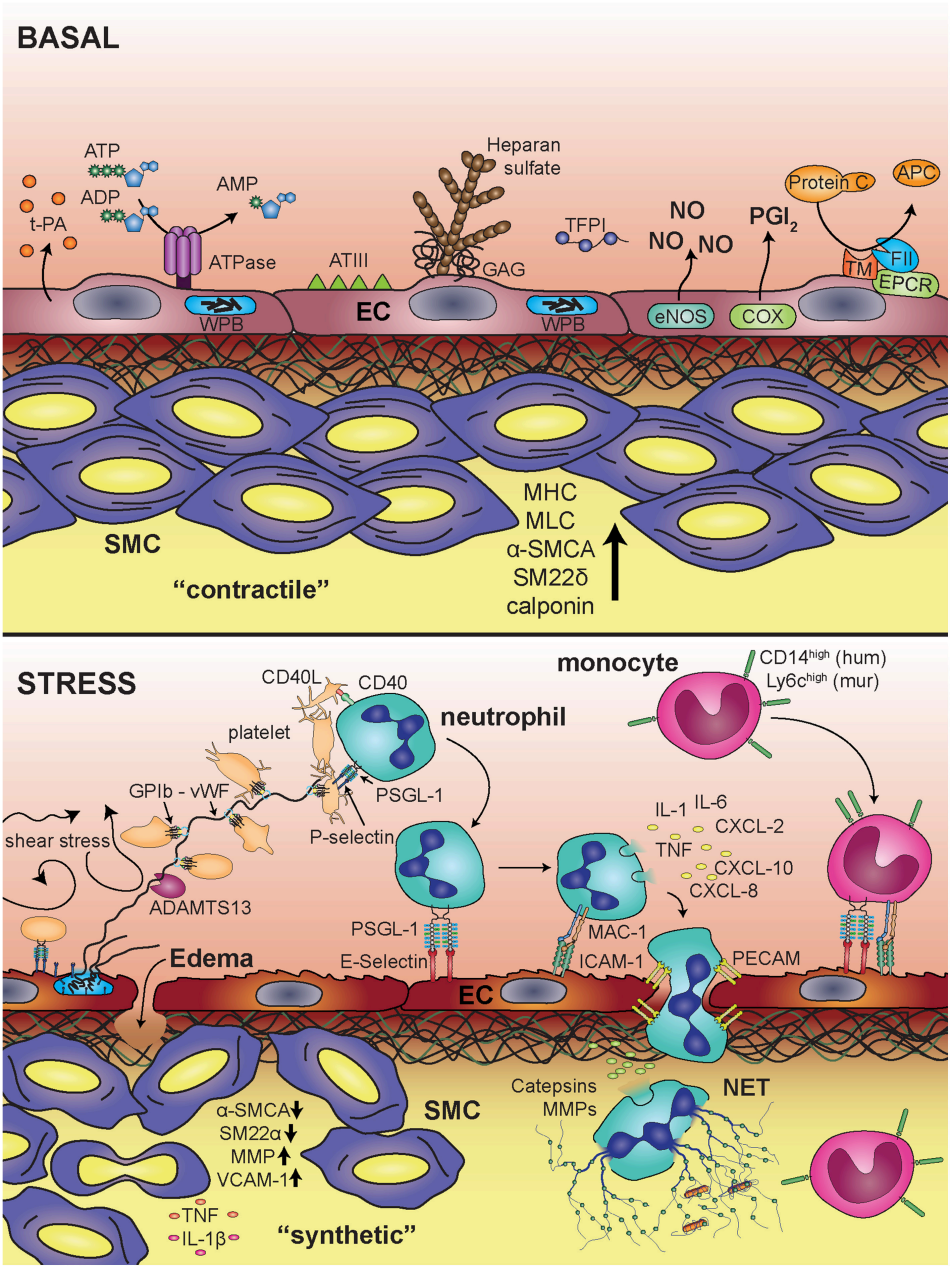
Blood vessel under basal conditions and upon inflammatory stimulation. Under basal conditions the endothelium provides an anti-thrombogenic surface via expression and production of tissue plasminogen activator (t-PA), ATPases, antithrombin III (ATIII), heparan sulfate, glycosaminoglycans (GAGs), tissue factor pathway inhibitor (TFPI), nitric oxide (NO), prostacyclin (PGI_2_), and endothelial protein C receptor (EPCR). Smooth muscle cells are in a “contractile” state, determined by expression of myosin heavy chain (MHC), myosin light chain kinase (MLCK),α-smooth muscle cell actin (α-SMCA), smooth muscle 22α (SM22α), and calponin. Upon inflammatory stress, endothelial cells release von Willebrand Factor vWF from Weibel Palade bodies (WPB), which triggers platelet string formation via glycoprotein Ib (GPIb). Furthermore, adhesion and transmigration of leukocytes is facilitated by expression of adhesion molecules, like E-selectin and intracellular adhesion molecule 1 (ICAM-1), which bind to PSGL-1 and Mac-1 on leukocytes, respectively. Activation of neutrophils leads to release of inflammatory mediators (IL-1, IL-6, TNFα, CXCL-2, CXCL-10, CXCL-8). Smooth muscle cells change their phenotype toward a “synthetic” state associated with decreased expression of α-SMCA and SM22α and increased expression of matrix metalloproteinases (MMP) and vascular cell adhesion molecule 1 (VCAM-1).

The endothelial cell lining of blood vessels represents a selective barrier between the blood stream and the surrounding tissue and exerts a variety of functions that contribute to hemostasis, and inflammatory responses that are related to coagulation (295). Many of these reactions are specific to their localization within the body as endothelial functions vary between different vascular beds. Under homeostatic conditions, endothelial cells constantly secrete nitric oxide, prostacyclin (in large vessels) as well as prostaglandin E2 (in smaller vessels) to suppress platelet adhesion and activation (Figure 6, upper panel) (4, 296). This is additionally supported by negatively charged glycosaminoglycans on the endothelial surface that prevent adhesion of platelets. The NF-κB signaling cascade has a key role in endothelial cells in response to stress situations (Figure 6, lower panel), as it is capable of regulating both pro-inflammatory and coagulatory responses, which are also prone to a significant level of crosstalk (297). In principal, all NF-κB signaling molecules are present in endothelial cells and their activation leads to a pro-adhesive and pro-coagulant phenotype with a concomitant reduction of the barrier function (298). *In vitro*, the strongest activators of NF-κB in endothelial cells appear to be TNFα and thrombin, but also other cytokines like IFNγ or IL-1β potently activate NF-κB in these cells. One major difference of thrombin- and TNFα-mediated NF-κB activation lies in their respective receptors. Thrombin binds to the extracellular terminus of PAR-1, a member of the G-coupled receptor superfamily, whereas TNFα binds to TNFR-1 and TNFR-2 (299, 300). Both pathways then converge at the level of the IKK complex (76, 301), yet interestingly, thrombin and TNFα appear to induce some overlapping but still differential target gene expression in endothelial cells (302). In addition, there appears to be a synergistic effect of TNFα and thrombin in regulating endothelial permeability (303). Important NF-κB target genes in endothelial cells are adhesion molecules such as intercellular adhesion molecule 1 (ICAM-1), vascular cell adhesion molecule 1 (VCAM-1), and E-selectin that mediate adherence of inflammatory cells including monocytes, neutrophils, lymphocytes, and macrophages to the vascular wall triggering extravasation into tissues (304–307). It has been shown that expression of a constitutively active form of IKKβ, the central activator of NF-κB, in endothelial cells drives full expression of these adhesion molecules in the absence of any cytokine stimulation, indicating that the IKK/IκB/NF-κB axis is essential and sufficient for the pro-inflammatory activation of the endothelium (308). However, in quiescent endothelial cells, the ETS-related gene (ERG) prevents NF-κB p65 binding to DNA, indicating that ERG may compete with p65 for DNA binding under basal conditions (309).

Besides classical activation of endothelial cells by various cytokines, they can also be activated by shear stress, meaning specifically a turbulent blood stream: Unidirectional, laminar shear stress actually limits endothelial activation and is associated with resistance to atherosclerosis (310, 311). In contrast, disturbed flow, such as turbulent or oscillatory conditions (e.g., at sites of vessel branching points, bifurcations, and curvatures) cause physical stress and subsequent pro-inflammatory gene expression that is associated with increased permeability of the cell layer (310, 311). Flow-induced endothelial cell activation is mediated via NF-κB and is integrin-and matrix-dependent (312). Recent studies indicate that focal adhesion kinase regulates NF-κB phosphorylation and transcriptional activity in response to flow (313). Another important aspect refers to the function of PECAM-1, which forms a mechanosensory complex with vascular endothelial cell cadherin and VEGFR2. Together, these receptors confer responsiveness to flow as shown in PECAM-1-knockout mice, which do not activate NF-κB in regions of disturbed flow. This mechano-sensing pathway is required for the earliest-known events in atherogenesis (314).

In addition to NF-κB-driven transcriptional responses to inflammatory states, endothelial cells also react to stress stimuli in other ways. The most prominent one of these is probably the fusion of specific secretory granules designated as Weibel-Palade bodies (WPB) with the cell membrane upon activation by various triggers such as thrombin or histamine. Exocytosis of these granules can also be induced by Toll-like receptors and other activators of the NF-κB pathway such as CD40L implying a role of NF-κB signaling molecules for the degranulation (315–319). Upon membrane fusion, the cargo of the vesicles is released, which includes several proteins that play a role in inflammation and thrombosis such as coagulation factor VIII, vWF, or P-selectin. The latter is exposed on the endothelial cell surface upon fusion of WPBs with the cytoplasmic membrane, triggering the adhesion of leukocytes. vWF is a large glycoprotein, which remains in a folded state in the microenvironment of WPBs, but is unfolded at the neutral pH of the blood circulation. This leads to the formation of ultra-large vWF multimers (ULVWF), which are able to bind platelets via interaction with GPIba or GPIIb (4). ULVWF multimers are subsequently cut by metalloproteinase ADAMTS13 on the endothelial surface providing a feedback mechanism to counteract platelet adhesion (320).

Endothelial cells modulate the balance between coagulation and fibrinolysis also by various other pathways: they counteract coagulation by binding of antithrombin III to the endothelial surface, release of tissue factor pathway inhibitor (TFPI), expression of thrombomodulin, activation of protein C, and synthesis as well as release of tissue plasminogen activator (t-PA) (296). These anti-thrombotic roles are often terminated by inflammatory diseases as for instance in endotoxemia and septic shock where a bacterial infection causes a pro-thrombotic state in endothelial cells by the activation of NF-κB (321). This has been demonstrated in a mouse model of sepsis, where endothelial-selective blockade of NF-κB via transgenic expression of a degradation-resistant form of IκBα resulted in decreased endothelial permeability, reduced infiltration of neutrophils and lower levels of thrombin-antithrombin complexes (322). Other mouse studies confirmed that endothelial NF-κB dampens the thrombomodulin-EPCR anticoagulation pathway (323). In addition to this systemic effect, NF-κB also serves as survival factor for endothelial cells themselves upon LPS-induced acute stress (324). This is also important for the maintenance of the endothelial barrier function as NF-κB inhibits endothelial apoptosis to ensure timely transition from barrier injury to recovery (325). Interestingly, endothelial expression of a degradation-resistant form of IκBα did not affect embryonic development, while endothelial cell-specific knockout of IKKβ resulted in increased embryonic lethality and endothelial apoptosis, which was at least in part mediated by kinase-independent functions of IKKβ (326).

A crucial role of endothelial NF-κB signaling has also been shown in mouse models of atherosclerosis where ablation of canonical NF-κB signaling by endothelial cell-specific deletion of NEMO or overexpression of a dominant-negative variant of IκBα protected ApoE-deficient mice from atherosclerosis induced by a Western-type diet (327). In general, atherosclerosis can be considered as chronic inflammatory disease of the vasculature, which is characterized by a complex crosstalk between different cell types, with endothelial cells constituting a crucial starting point of a vicious cycle, wherein NF-κB activation does not only lead to the expression of adhesion molecules that bind leukocytes, but also causes secretion of inflammatory mediators, which activate smooth muscle cells. This leads to vascular remodeling resulting in the plaque formation and narrowing of the vessel lumen. Furthermore, endothelial cells could undergo a reprogramming process toward a mesenchymal phenotype, designated as endothelial-mesenchymal transition, which is characterized by the expression of smooth muscle actin, various fibroblast markers and collagen (328). This phenotypic shift was reported to be involved in endothelial dysfunction during atherosclerosis. It can be triggered by cytokines such as TGFβ or IL-1, high glucose levels or pressure overload, as well as oxidized LDL (329–331).

## Vascular Smooth Muscle Cells

Vascular smooth muscle cells (SMCs) are important players in both inflammatory and thrombotic processes. In general, arteries and veins consist of three layers, the tunica adventitia, largely constituted by connective tissue and fibroblasts, the tunica media mainly containing vascular smooth muscle cells and the tunica intima. Separated from the media by the internal elastic membrane, the intima consists of loose connective tissue intermingled with few SMCs, that is covered by a monolayer of endothelial cells resting on a basal membrane. The main function of SMCs in a blood vessel is to regulate the caliber. In a normal vessel, SMCs are in the contractile phenotype (Figure 6). They have very low cell division rates, a very restricted migratory behavior and express high levels of contractile proteins, such as myosin heavy chain, myosin light chain kinase, calponin, smooth muscle actin, and SM22α. Under conditions of inflammation, SMCs gain plasticity—their phenotype can change from contractile to synthetic; they rearrange their cytoskeleton, loose expression of contractile proteins, and regain their ability to proliferate and migrate. This phenotypic switch is central to many vascular diseases, such as atherosclerosis, re-stenosis, and vascular aging (332). The important role of SMC in stabilizing the cytoskeleton is highlighted in patients with mutations in ACTA2 encoding for smooth muscle actin or its promoter, leading to a higher risk for coronary disease (333). In atherosclerotic plaques, which represent chronically inflamed parts of arteries, SMCs reside predominantly in the superficial parts of lesions. They are mainly locally derived from the vessel wall (334). Phenotyping of the cells within the plaques revealed sizeable populations of SMCs without contractile proteins (335). Of note, also macrophages can express SMC genes such as smooth muscle α-actin and SM22α. Thus, SMC marker–positive cells can be derived from cell types other than SMCs and SMC marker–negative cells can be SMC-derived. Finally, even cells that are positive for CD68—the common macrophage marker, may not be macrophages as SMCs can undergo a cellular transition toward macrophage-like cells while simultaneously losing some of their SMC characteristics. This has been elucidated in more detail by genetic cell tracing approaches, which could show that more than 80% of SMC-derived cells within atherosclerotic lesions lack SMC markers that are commonly used in immuno-histochemical stainings, and that more than 30% of SMC-derived cells express conventional macrophage markers (336, 337). This means that many studies might have misinterpreted cellular markers and that probably many disease processes attributed to macrophages are in fact driven by SMCs that converted their cellular program. An important aspect is that SMC-derived macrophage-like cells are apparently less efficient in phagocytosis of deposits and apoptotic cells within the plaque as compared to “real” macrophages, which exacerbates necrotic core formation rendering the plaque unstable and prone for rupture (338, 339).

Anyway, these cells produce fibrous caps, and SMCs are an important source of collagen (340), which activates platelets, when endothelial cells are lost due to plaque rupture or erosion. The downregulation of SMC contractile genes such as SM22α is a typical phenomenon of atherosclerotic lesions (341). Interestingly, SM22α suppresses NF-κB signaling pathways under inflammatory conditions (342).

SMCs express multiple NF-κB family members and two inhibitor proteins, IκBβ and IκBα. In normal vessels SMCs display no basal NF-κB activity but the latter is readily induced in SMCs within atherosclerotic lesions. Interestingly, exposure to inflammatory cytokines induces prolonged NF-κB activation because of a sustained decrease in the inhibitory subunit IκBβ (343). TNFα appears as a crucial factor for the progression of atherosclerotic lesions as shown in TNFα/ApoE double knock-out mice, which display reduced thickness of vascular walls and reduced sizes of atherosclerotic lesions (344). TNFα binds to TNF receptors expressed on SMCs (345), which then triggers NF-κB via the classical activation pathway. This induces the expression of the pro-coagulatory tissue factor gene (346), as well as pro-inflammatory and matrix-remodeling genes such as MCP-1, matrix metalloproteinase-3 and−9 (MMP3 and MMP9), VCAM-1, and IL-1β, and furthermore potently downregulates SMC contractile genes (smooth muscle actin, SM22α, smooth muscle myosin heavy chain) (347). TNFα decreases expression of these contractile genes through induction of Krüppel-like transcription factor 4 (Klf4), a known regulator of SMC differentiation (348), which seems to be a target gene of NF-κB, based on specific binding sites in its enhancer region (337).

Even though a direct link between the downregulation of SMC contractile genes, NF-κB signaling and an increased risk for plaque rupture and arterial thrombosis has yet not been made, it is clear that elucidating mechanisms of phenotypic changes of SMCs in the course of inflammation seems to be a key in understanding many vascular diseases and may offer new therapeutic approaches.

## Neutrophils

Neutrophils are the most abundant leukocyte fraction in humans with a rapid turn-over controlled by constitutive (spontaneous) apoptosis within 24–48 h after release from the bone marrow. Their life-span is markedly extended during inflammatory reactions and coupled to neutrophil activation to promote the inflammatory response (349). Since both, cell survival and pro-inflammatory activation are regulated by NF-κB, this transcription factor is central to neutrophil function and shows a unique expression pattern distinct from other leukocyte subsets (350, 351). In unstimulated neutrophils, NF-κB and in particular IκBα are not restricted to the cytosol as in most other cells but show abundant localization to the cell nucleus, with nuclear IκBα being regarded as a protective mechanism preventing the NF-κB-dependent expression of pro-inflammatory and anti-apoptotic genes (351). Furthermore, the IKK complex is partially localized to the nucleus. Upon neutrophil activation, IKKβ and NEMO are phosphorylated in the cytosol as well as the nucleus while IKKα is entirely lost from both compartments. The subsequent IκBα degradation and phosphorylation of RelA at serine 536 then promote NF-κB target gene expression (352).

Functional dimers of p50 (NFκB1), p65 (RelA), and/or c-Rel are detectable in neutrophils, and their activity is induced by a vast variety of pro-inflammatory mediators (353). While the majority of stimuli including TNFα and LPS trigger DNA binding by p50 and RelA (354), distinct agonists such as G-CSF selectively induce c-Rel activity (355). The first studies showing p50/RelA activation in neutrophils by pathogens, revealed the process of phagocytosis as an important trigger (356, 357). Subsequently, engagement of toll-like receptors (TLRs) by microbial products was identified to regulate NF-κB activity in neutrophilic granulocytes (358), with agonists of TLR4 (359, 360), TLR2 (361, 362) but also TLR7/8 (363) and TLR9 (364, 365) serving as important activators. Apart from TLRs, other pathways for sensing pathogen- or damage-associated molecular patterns [involving e.g., CIRP or Sox2 (366, 367)], as well as pathogen recognition *via* Fc receptors (368), were more recently identified to control neutrophil activation via NF-κB.

Neutrophil adhesion in the course of an inflammatory reaction is primarily mediated by activated β2 integrins (Mac-1: CD11b/CD18). Integrin binding or aggregation reportedly promotes NF-κB activation to enhance pro-inflammatory and anti-apoptotic gene expression (369). Furthermore, the β2 integrins may function as co-stimulatory signals for cytokines like GM-CSF and IL-8 to activate NF-κB when neutrophils are attached as opposed to suspended (370). Also myeloperoxidase released by these cells may bind to CD11b/CD18 and enhance the activation of NF-κB (371). Engagement of other integrins such as α9β1 by the respective ligand (VCAM-1 on endothelial cells) results in a comparable effect on NF-κB function (372, 373).

In the context of hemostasis and thrombosis, activated platelets expose CD40L at their surface which binds to neutrophil CD40 thereby inducing NF-κB target gene expression via the alternative activation pathway (374). Interestingly, platelet-derived microparticles reportedly transfer glycoprotein IIb/IIIa receptors onto neutrophils, which co-localize with β2-integrins and enhance NF-κB activation (375). Apart from platelets, coagulation factors and derived fragments may function to guide neutrophil activation and extend the neutrophil life-span via NF-κB transcriptional activity. For example, fibrinogen triggers IκBα degradation and NF-κB activation by binding to CD11b/CD18 molecules (376). In addition, the F1 and F2 fragments which are released upon prothrombin processing are known to induce NF-κB activity in neutrophils (377). Furthermore, regulators of plasmin activation (PAI-1 and uPA) may potentiate the polymorphonuclear (PMN) cell response to pro-inflammatory stimuli with respect to NF-κB activation (378).

Moreover, ROS have been implicated in the signaling pathway leading to NF-κB activation (379). However, the impact of ROS such as hydrogen peroxide (H_2_O_2_) generated at inflammatory sites has been subject to extensive debate and contradictory reports with respect to NF-κB activation in neutrophils. Direct exposure of neutrophils to H_2_O_2_ does not result in NF-κB activity. In contrast, the effect of LPS- or TNFα stimulation are abrogated by H_2_O_2_ resulting in decreased IκBα degradation and NF-κB translocation (380, 381). Similarly, when intracellular levels of ROS (superoxide and hydrogen peroxide) are increased by inhibition of catalase or the mitochondrial electron transport chain, the pro-inflammatory activation of NF-κB is inhibited (382–384). However, distinct approaches to raise intra- or extracellular superoxide levels (based on paraquat, nickel or combinations of xanthine oxidase and hypoxanthine or lumazine) showed a promoting rather than inhibiting effect on NF-κB activation (385–387). The controversial results may indicate that ROS regulation of NF-κB activity at inflammatory sites is more complex than previously thought and that ROS may exert both, pro- and anti-inflammatory effects. While low doses of H_2_O_2_ seem to trigger NF-κB activation, high oxidative stress does not alter or even adversely affect the NF-κB status (388, 389). Comparably, myeloperoxidase was recently reported to engage in a negative feedback loop of NF-κB downregulation to dampen the pro-inflammatory cytokine response (390). Other inhibitors of NF-κB activation in neutrophils include nitric oxide (391, 392), complement factor C5a (393), and prostaglandin D2 (394).

The target genes regulated by NF-κB in neutrophils can be grouped according to the three major functions of mediating cell adhesion, promoting inflammation, and inhibiting neutrophil apoptosis. In contrast, phagocytosis does not seem to be dependent on NF-κB (395). The induction of integrin CD11b expression requires p65 and promotes the firm adhesion and transmigration of neutrophils (395, 396). Activated PMNs secrete a multitude of pro-inflammatory mediators. Among the NF-κB regulated genes are the cytokines TNFα, IL-1, IL-6 (397, 398), the chemokines CXCL-2,−8, and−10 (360, 387, 397) as well as the TLR4 co-receptor CD14 (399) and the neutrophil gelatinase-associated lipocalin (400). Of interest, NF-κB activation also promotes microparticle release from PMNs (401). While NF-κB is known to exert a negative feedback regulation by inducing transcription of its inhibitor IκBα, an additional feedback mechanism has been identified in neutrophils: Expression of miR-9 is controlled by NF-κB and serves to inhibit the NFκB1 transcript (193).

Importantly, the balance between neutrophil production, survival and cell death is regulated by NF-κB. The mobilization of neutrophils from the bone marrow is subject to control by p50/p65 and seems to involve the NF-κB induced expression of the transcription factor C/EBPα (402, 403). While NF-κB is known to further support neutrophil survival and block spontaneous apoptosis, it may—in turn—facilitate cell death via neutrophil extracellular trap (NET) formation. Thus, NETosis is abrogated in the presence of NF-κB inhibitors such as BAY 11-7082 and Ro 106-9920 (404, 405), although it has to be stated that these inhibitors may also have NF-κB independent effects.

In the context of hemostasis and thrombosis, it was shown that activated platelets promote NET formation by a variety of signals including HMGB1 which induces neutrophil autophagy and subsequent expulsion of DNA NETs (229). It was proposed that autophagy constitutes an essential second step required to trigger NETosis after the initial pro-inflammatory priming of neutrophils (406). Thus, in addition to its role in the inflammatory activation of neutrophils, NF-κB may contribute to further steps of NET induction, as it exerts context-dependent effects on autophagy (407). Importantly, NETs seem to provide a scaffold for platelet, erythrocyte, tissue factor and fibrin deposition, which reportedly promotes arterial and venous thrombosis (227, 408–412). NET-exposed histones as well as neutrophil proteases such as elastase and cathepsin G are known to further enhance platelet activation and to degrade inhibitors of coagulation (413, 414). The detrimental role of NETs in thromboembolic disease has specifically been addressed in the cancer setting (415, 416). Tumor cells were shown to directly trigger NET formation or prime platelets to promote NETosis which results in further platelet activation and release of tissue factor (417, 418). In addition, this process of NET-associated cancer thrombosis is enhanced by tumor-cell derived microparticles (419). Most recently, clinical evidence is corroborating the association between NET formation and thrombosis in cancer patients (420, 421).

The control of neutrophil apoptosis is central to the inflammatory reaction as well as resolution and is primarily dependent on the NF-κB mediated expression of anti-apoptotic genes such as Bcl-x(L), A1, and A20 (363, 422). Thus, unstimulated neutrophils are characterized by the predominant presence of IκBα in the cell nucleus which inhibits NF-κB activity and allows for spontaneous apoptosis and rapid cell turn-over. When the nuclear accumulation of IκBα is artificially increased or when NF-κB activation is blocked, the constitutive apoptosis is accelerated (423, 424). In contrast, the pro-inflammatory activation of neutrophils by e.g., TNFα, LPS, type I interferons, or IL-1β results in IκBα degradation in the cytosol and nucleus and the subsequent liberation of NF-κB to prevent apoptosis (349, 425–428). The signaling pathway of TNFα for NF-κB activation is best characterized in this context. TNFα has a bimodal influence on the rate of neutrophil apoptosis *in vitro*, causing early acceleration and late inhibition when NF-κB dependent expression of anti-apoptotic proteins is achieved (429). TNF receptor 1 (TNFR-1) mediates activation of PI3 kinase and PKC-delta which results in assembly of the TNFR-1-TRADD-RIP-TRAF2 complex required for anti-apoptotic signaling (430). Apart from pro-inflammatory cytokines, it is the integrin-mediated adhesion and transmigration of neutrophils, which substantially enhances NF-κB mobilization and thereby promotes cell activation and survival in the situation where neutrophils extravasate from blood into tissue to engage at inflammatory sites (373, 431). Importantly, since hemostasis is closely linked to inflammation, the factors of coagulation and fibrinolysis also critically contribute to the localized activation and enhanced life-span of neutrophils. For example, binding of neutrophil surface integrin to fibrinogen activates NF-κB and delays apoptosis (376), and the release of prothrombin fragments or activation of uPA/PAI-1 may similarly enhance NF-κB activity (377, 378).

The shift in balance from spontaneous apoptosis to cell survival is reflected in the expression levels of pro- and anti-apoptotic mediators in PMNs. While pro-apoptotic proteins such as Bad, Bax, Bak, and Bik show stable expression and long half-lives, the NF-κB induced anti-apoptotic regulators like A1 and Mcl-1 are comparably short-lived and seem to transiently tilt the balance toward survival as long as NF-κB remains active (363, 364, 432).

The resolution of these processes at later phases requires the down-modulation of NF-κB activity by the re-expression of IκBα (350) and the induction of counter regulators such as suppressor of cytokine signaling 3 (SOCS3) (433). Failure to downregulate NF-κB results in the inappropriate survival of neutrophils, chronic inflammation, and tissue damage which is associated with neutrophil-mediated inflammatory disorders such as sepsis, rheumatoid arthritis and acute lung injury (349, 434, 435). Furthermore, sustained neutrophil activation and survival via the NF-κB pathway have been shown to promote tumor progression and metastasis by providing a pro-tumorigenic and pro-angiogenic environment (436, 437).

## Monocytes

Monocytes contribute essentially to pro-inflammatory immune responses in general. In parallel with neutrophils, monocytes are produced in high numbers in the bone marrow as a response to infections and diseases and are responsible for driving inflammation (438). In addition, monocytes are the main source of circulating TF (439). The myeloid linage gives rise to a variety of functionally diverse cell types and is therefore in need of a tightly regulated differentiation program, which is partly built around the NF-κB pathway (440, 441).

Overall, monocytes can be divided into several subsets. Within the human monocyte compartment, three distinct monocyte populations can be defined according to their expression of CD14 and CD16. Monocytes positive for CD14 and negative for CD16 are termed classical monocytes (CMs) and are the most abundant subset within the human circulation followed by intermediate monocytes (IMs), defined by CD14^++^CD16^+^ expression and non-classical monocytes (NCMs), which are CD14^+^CD16^++^. The differentiation of monocytes from classical to intermediate and the non-classical phenotype is a linear process. In humans, classical monocytes are the first subset to emerge from the bone marrow, followed by differentiation into intermediate and non-classical monocytes (442). In addition, differentiation of monocytes is connected to cellular aging as NCMs display clear markers of cellular senescence including reduced telomere length and reduced numbers of Ki67-positive cells (443).

CD16^+^ monocytes overall are more proinflammatory and more procoagulant. In general IMs and NCMs display increased protein levels of p65 (443). When healthy volunteers are infused with low-dose LPS, CD16^+^ monocytes respond with upregulation of IL-6 and IL-8 which could not be observed in CD16^−^ monocytes (444). Furthermore, *in vitro* IMs reacted to the alarmin IL-33 with an upregulation of TF via an NF-kB dependent pathway, a pathway probably active also in patients with atherosclerosis as monocyte-derived microvesicles positive for TF were correlated with IL-33 plasma levels (445).

In contrast to human monocytes, mouse monocytes are classified into pro-inflammatory and patrolling monocytes. Even though there are differences between mouse and human monocytes, monocyte subsets within the two species are broadly conserved (446). Pro-inflammatory monocytes are characterized by high expression of Ly6c. This subset of monocytes is strongly associated with encountering infections and driving inflammation. Expression of inflammatory cytokines, chemokines, and ROS production have been observed during heavy recruitment to inflamed tissue in various models (438). Definition and characterization of the Ly6c^low^ CXCR1^hi^ patrolling monocyte subset appears to be more complicated. Their exact role during homeostasis is not completely understood, but it is known that they show features for tissue remodeling and restoration (447). Further they tend to express anti-inflammatory mediators, like IL-10 and arginase (ARG1) (448), which suggest a counterbalancing role against the pro-inflammatory subset.

The balance of murine subsets has been suggested to be mainly defined by GM-CSF and M-CSF stimuli (449, 450), which are both triggering the NF-κB pathway (31, 451). NF-κB itself generates a positive feed-back loop to produce M-CSF (452).

Monocytes require NF-κB for differentiation but also accumulate NF-κB in their cytoplasm during maturation in order to guarantee a rapid NF-κB response upon activation (440). TNFα, which is secreted very early, represents one of the most prominent inflammatory genes, which is induced by the accumulated NF-κB reservoir, subsequently triggering a pro-inflammatory program of monocytes, or macrophages in an autocrine manner.

Importantly, monocytes require growth factors, like M-CSF, not only for differentiation but also for survival. Many of these stimuli are dependent on NF-κB signaling, suggesting a chronical dependence of monocytes on this pathway for survival. This has originally been demonstrated by studies using the NF-κB inhibitor pyrrolidine dithiocarbamate (451, 453) and could be confirmed with other NF-κB inhibitors when studying human monocyte-derived dendritic cells. In this study a role of NF-κB was demonstrated for survival, cytokine production and differentiation (454). More recently, it has been revealed that monocytes require autonomous TNFα to achieve function, survival and maintenance of the Ly6c^hi^ subset in an experimental autoimmune encephalomyelitis (EAE) model (455). These findings indicate a critical regulatory function for NF-κB in the autonomous loop of monocytes, as TNFα is driven by NF-κB and, in turn, is a strong inducer of NF-κB by itself (456, 457). Monocyte-specific constitutive activation of NF-κB resulted in a more severe pathogenicity within the EAE model and demonstrated increased levels of inflammatory monocyte-associated cytokines (458). Future studies are required to determine the potential regulatory mechanism of NF-κB in this context.

Interestingly, mouse studies using myeloid-specific deletion of the central NF-κB activator IKKβ revealed an interesting effect on macrophage polarization. Deletion of IKKβ resulted in a shift toward the inflammatory M1 phenotype both in an infection- and a tumor model, indicating a role of IKKβ and NF-κB for polarization toward the M2 phenotype, which decreases inflammation and fosters tissue repair (459, 460).

A disease, where monocytes play a crucial role is atherosclerosis. This complex disorder is orchestrated by multiple variables and cell types, but is fundamentally dependent on infiltrating monocytes (461). In this context, macrophage-specific deletion of IKKβ resulted in an aggravation of atherogenesis in one study (462), while a similar experimental set-up used in another study showed reduced lesion area (463). A protective effect of macrophage IKKβ in the context of atherosclerosis would be in line with the above-mentioned notion that IKKβ deletion or inhibition leads to a shift toward to the M1 phenotype, which is known to drive atherosclerosis. This concept is also supported by the observation that transgenic mice with macrophage-specific upregulation of p65 exhibited reduced atherosclerotic lesion formation and foam cell development (464). In contrast to that, another study with myeloid cell-specific IκB deletion (expected to result in elevated p65 activity) claimed an increase in atherosclerosis (465). Thus, a clear picture on the role of macrophage-specific NF-κB in atherogenesis is still lacking. For atherosclerosis, it is arguable that enhanced NF-κB expression may delay foam cell formation but might have severe consequences in a later stage of the diseases. For instance, increased NF-κB signaling in monocytes also results in a more pronounced expression of tissue factor (466), a critical variable in the pathology of atherothrombosis (467). The relevance of monocyte-derived tissue factor for thrombus formation has been demonstrated in an elegant study of impaired blood flow by von Brühl et al. (227). The authors identified neutrophils and monocytes to be the major leukocyte populations responsible for thrombus development. They found that, besides neutrophil-mediated NETosis, monocyte-derived tissue factor is critical for fibrin generation within the thrombus and contributes fundamentally to thrombus development. This is in line with findings showing a correlation of monocytic NF-κB activity with the occurrence of deep vein thrombosis (DVT) in cancer patients (468). A similar concept has already been suggested based on experimental results describing the necessity of p50 in the pathogenesis of deep vein thrombosis (469).

In conclusion, we know that the NF-κB pathway is involved in multiple aspects of monocyte differentiation and activation, which makes it difficult to distinguish the role of NF-κB in each individual stage of monocytes. It will require elegant inducible gene-manipulation strategies to answer these questions but considering the major influence of NF-κB on monocyte behavior, it might open doors for therapy of a broad spectrum of inflammatory diseases.

## Clinical Aspects: Sepsis as an Example of an Acute Thrombo-Inflammatory Disease State

**Figure 7:**
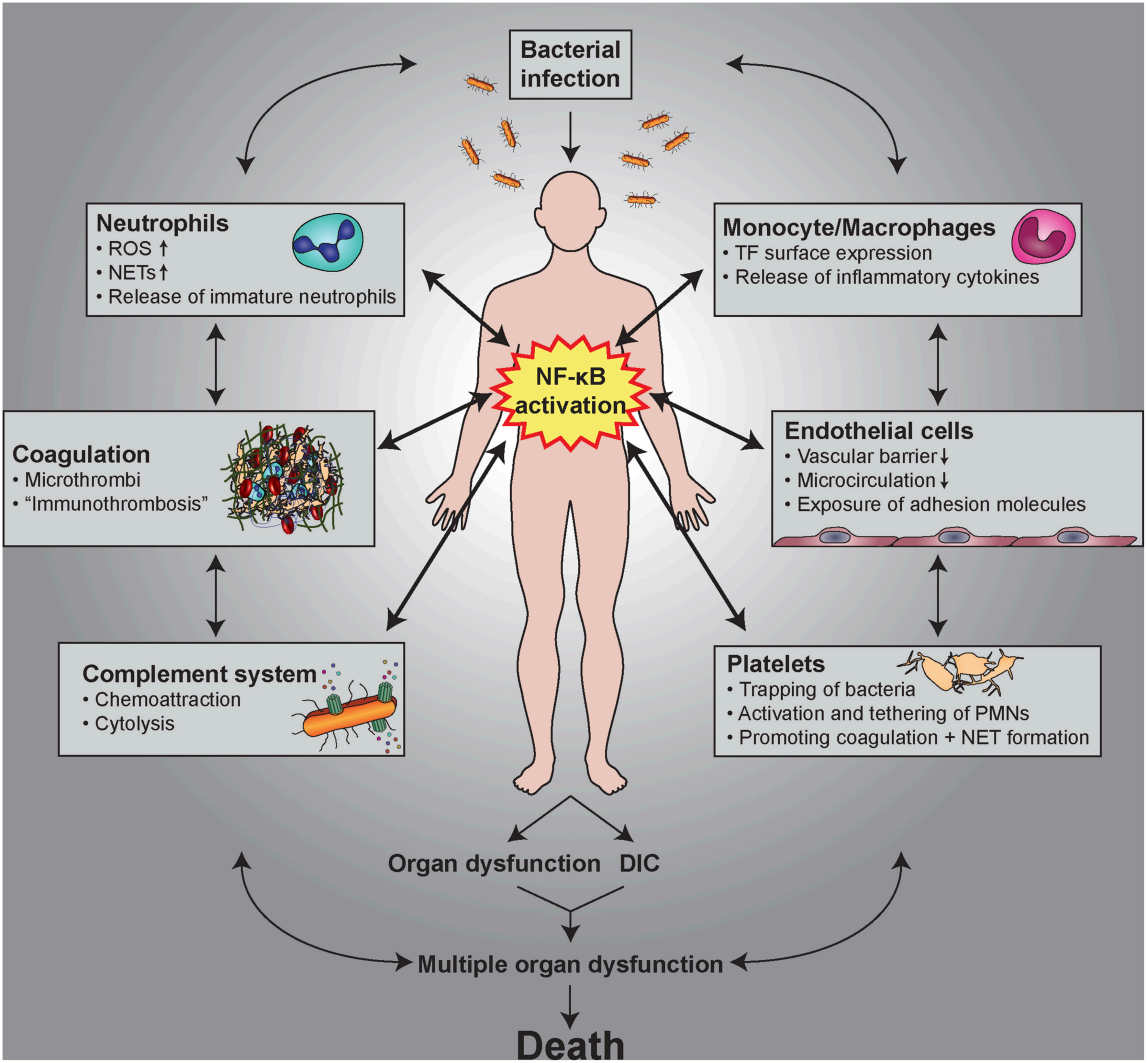
Hallmarks of sepsis as a thrombo-inflammatory disease. Multiple, complex interactions between monocytes/macrophages, endothelial cells, platelets, the complement system, coagulation, and neutrophils are found under septic conditions. Activation of NF-κB causes not only the release and/or the generation of a multitude of pro-inflammatory mediators, but also the induction of pro-coagulatory mechanisms, which lead to the clinical signs and symptoms of sepsis.

The vasculature and cells of the circulatory system react in a complex manner to inflammatory stress including various feedback circuits and cellular crosstalk coordinating a common systemic response in order to protect the host (Figure 6). However, dysregulation of this subtle balance between physiological inflammation and coagulation causes chronic inflammation and pathological thrombosis. Sepsis is a prime example of such a dysregulated response, which can lead to life-threatening conditions caused by an overshooting host defense (470). In general, the term sepsis denotes a systemic inflammatory response to infection. It is initiated by the activation of innate immune cells via pathogen-associated molecular patterns (PAMPs), such as lipopolysaccharide (LPS), microbial peptides, cell wall components, or nucleotides, which trigger various receptors on the host cells: C-type lectin receptors; Toll-like receptors (TLRs); RIG-I like receptors, as well as nucleotide-binding oligomerization domain–like receptors (NOD-like receptors). These and similar receptors can also stimulated by so-called danger associated molecular patterns (DAMPs) or “alarmins,” which include various cytosolic proteins, extracellular RNA or DNA that could all be released from damaged cells. In this way, necrosis or physical cell damage as it occurs in course of poly-traumas can trigger sepsis-like processes (commonly termed systemic inflammatory response syndrome, SIRS) in the absence of any infectious pathogen (471). Finally, most of these pattern recognition receptors activate NF-κB, which causes the expression of inflammatory cytokines like IL-1β or TNFα. Since these cytokines are both target genes and triggers of NF-κB, positive feedback loops are initiated, which result in a so-called “cytokine-storm” (472). Furthermore, activation of NF-κB causes not only the release and/or the generation of a multitude of pro-inflammatory mediators, but also the induction of pro-coagulatory mechanisms, which altogether lead to the clinical signs and symptoms of sepsis as well as disseminated intravascular coagulation (DIC) and multiple organ dysfunction (473) (Figure 7). The latter is basically caused by widespread thrombus formation in capillaries and reduced blood pressure causing tissue hypoperfusion. The disseminated coagulation can be explained by NF-κB-mediated upregulation of tissue factor (F III) and F VIII in combination with a reduction of anticoagulatory mechanisms such as Tissue Factor Pathway Inhibitor (TFPI), antithrombin, or thrombomodulin (471). Moreover, inflammatory activation of neutrophils triggers the formation of NETs, which exert not only anti-microbial functions by trapping and killing bacteria, but also initiate the contact pathway of coagulation via F XI and XII (474, 475). Various components of NETs like histones and proteolytic constituents have been identified as crucial regulations of coagulation, which contribute to development of end-organ damage (413). Collaborative interactions between NET-derived histone H4, platelets and inorganic polyphosphates are able to promote disseminated coagulation intendent of the invading pathogen (8). The diminished oxygen supply caused by microvascular thrombi results in deregulation of mitochondria function, which leads to increased formation of ROS thereby aggravating tissue damage and contributing to the release of danger signals.

Extensive formation of thrombi in the microcirculation causes systemic depletion of coagulation factors and platelets resulting in increased bleeding events at other sites of the organism—a phenomenon generally designated as “coagulopathy.”

This imbalance is not only observed in coagulation—also inflammatory processes are affected. Due to strong, overshooting inflammatory responses in the first phase, counter-acting feedback-mechanism often become predominant at a later stage of the disease resulting in immunosuppression associated with increased risk for secondary or opportunistic infections. Attempts to understand the complex pathogenesis of sepsis included low-dose infusion of LPS into healthy volunteers (476). This revealed that LPS activates the endothelium and the coagulation system, as well as fibrinolysis, accompanied by a pro-inflammatory response (476, 477). Similar to LPS, infusion of the cytokine TNFα into healthy volunteers exerted not only pro-inflammatory actions, but also activated the coagulation cascade (478, 479).

Given the importance of NF-κB for the initiation of the vicious circle of sepsis, its inhibition has often been considered as an interesting therapeutic approach to treat or prevent overshooting immune responses (480). This notion is supported by different animal models of sepsis showing a beneficial effect of NF-κB inhibition (472, 481). However, blocking NF-κB activity is also accompanied by reduced host defense and thus elimination of pathogens—and is therefore contraindicated at the late state of sepsis. Thus, the right balance between positive and negative effects of NF-κB inhibition or the correct timing of blocking NF-κB have not been found, yet. This is reflected by various clinical trials blocking NF-κB or associated inflammatory pathways by treatment with anti-inflammatory substances (as listed in Table 3). These substances included glucocorticoids, which inhibit the NF-κB pathway, as well as non-steroidal anti-inflammatory drugs (NSAIDs) such as acetylsalicylic acid (ASA), which do not only block the synthesis of inflammatory mediators but also inhibit the activity of IKKs (501). Interestingly, ASA and its active metabolite salicylic acid (SA) exert both anti-inflammatory (502) and anti-coagulatory actions (503) and SA naturally occurs in the human body due to up-take of plant-based food and endogenous production (504). Furthermore, a number of antioxidants has been investigated, which indirectly inhibit the NF-κB activation pathway, including vitamin C, vitamin E, β-carotene, N-acetylcysteine, selenium, or omega-3 fatty acids (505–510). However, clinical trials with these antioxidants failed to show any beneficial effect in sepsis (496–500). On the other hand, beneficial effects of anti-inflammatory agents have been reported in a recent systematic meta-analysis showing that anti-TNFα treatment of septic patients slightly reduces mortality with an odds ratio of 0.91 (482). Furthermore, the relevance of LPS as trigger of sepsis could be underlined by studies applying extracorporeal endotoxin elimination devices with promising results (511).

**Table 3:** Clinical studies targeting the thrombo-inflammatory axis of sepsis.

**Agent**	**Short description**	**References**
Anti-TNFα	Reduction of mortality (OR 0.91)	(482)
Glucocorticoids	Reduction of mortality (OR 0.87)	(483, 484)
Ibuprofen (NSAID)	Improvement of biomarkers, no significant effect on mortality	(485)
Acetylsalicylic acid (ASA)	Lower mortality suggested; large trial still ongoing	(486–488)
Atorvastatin	Lower IL-6 levels implying anti-inflammatory effects; however, no clear effects on survival	(489)
Atorvastatin	Reduction of conversion to severe sepsis from 24 to 4%	(490)
Rosuvastatin	No effect in sepsis-induced ARDS	(491)
Azithromycin	Sepsis-induced ARDS: significant survival improvement (OR 0.38), immune-modulatory effect assumed	(492)
Edaravone (radical scavenger)	Reduction of mortality from 30 to 13% in septic peritonitis	(493)
Antithrombin III	No reduced mortality, but increased risk of bleeding (RR 1.58)	(494, 495)
Antioxidants	No beneficial effects of vitamins C and E, β-carotene, N-acetyl-cysteine, selenium, omega-3 fatty acids	(496–500)

Nevertheless, the various clinical trials on NF-κB inhibition in sepsis underline the complex role of NF-κB in immune defense, inflammation and coagulation and the difficulty to find the right timing or regimen of treatment. However, concepts of dampening NF-κB activity appear very promising in thrombotic diseases that are characterized by rather low-grade chronic inflammation. This was demonstrated in a recent large clinical trial applying anti-IL-1β antibodies in patients with atherosclerosis and a prior myocardial infarction. The anti-inflammatory effect could be shown by dose-dependent reduction of the CRP level with was associated with an decreased risk to develop a second infarction, non-fatal stroke or cardiovascular death (512). However, as expected anti-IL1β treated patients had a higher risk of infections.

Overall, it is clear that inflammatory processes and thrombotic events are tightly linked on many different levels and that the NF-κB signaling pathway plays a fundamental role in the molecular and cellular linkages. Since NF-κB itself is a central hub in this network of reactions, an unspecific inhibition of this transcription factor might cause unwanted side-effects or be less efficient due to complex feedback circuits. However, considering the diversity of the intracellular as well as intercellular signaling networks that are built around NF-κB, targeting more specific connections between inflammation and coagulation might be very promising to reduce thrombotic morbidities that are associated with numerous chronic inflammatory diseases.

## Author Contributions

MM wrote major parts of the manuscript, with an emphasis on endothelial cells, designed figures, and contributed to the overall conception. MS contributed major parts of the platelet- and megakaryocyte section and designed figures. CB wrote the part on neutrophils. BH contributed to the endothelial cell part. CS contributed to the sepsis section and summarized clinical trials targeting inflammation in sepsis. HD wrote major parts of the monocyte/macrophage section. PH wrote major parts of the monocyte/macrophage section. JB performed bioinformatics analysis and designed Figure 4. PP wrote major parts of the smooth muscle cell section. AA contributed major parts to the platelet and megakaryocyte section. JS made the concept for the manuscript, wrote the parts on NF-kappa B, the NF-kappa B signaling pathways, contributed major parts to the sections on endothelial cells, smooth muscle cells, and monocytes, and wrote major parts of the sepsis section, designed Figures 1, 2 as well as parts of Figures 3, 6 and the concept for Figure 4 and also created Tables 1, 2 and contributed to Table 3.

### Conflict of Interest Statement

The authors declare that the research was conducted in the absence of any commercial or financial relationships that could be construed as a potential conflict of interest.
